# Environmental epidemiology in a crossfire

**DOI:** 10.1186/s12940-021-00776-1

**Published:** 2021-08-19

**Authors:** Ruth A. Etzel, Philippe Grandjean, David M. Ozonoff

**Affiliations:** 1 Milken Institute School of Public Health, The George Washington University, D. C, Washington, 20052 USA; 2 Harvard T.H. Chan School of Public Health, Department of Environmental Health, Boston, MA 02115 USA; 3 Department of Public Health, University of Southern Denmark, Odense, Denmark; 4 Department of Environmental Health, Boston University School of Public Health, Boston, USA

**Keywords:** Conflict of interest, Doubt, Environmental epidemiology, Infodemic, Policy, Public health, Research integrity

## Abstract

Two tendencies have emerged in environmental epidemiology that hamper the translation of research findings into prevention of environmental hazards. One is the increased focus on highlighting weaknesses of epidemiology research that is clearly meant to explain away the research conclusions and weaken their possible implications for interventions to control environmental hazards. Another is the voluminous amount of information sharing that involves a substantial amount of misinformation, as part of the ongoing infodemic. In this light, the appearance of the catalogue of doubt-raising strategies, indeed the worst practices of scientific inference, is good news. Collected under the auspices of the International Network for Epidemiology in Policy, it serves to illustrate the range of possible (and impossible) forms of critique that may be raised on behalf of vested interests or other groups who for some reason disagree with the epidemiological conclusions. We believe that this systematic list will be useful in our field and help to identify critiques of policy options that are hidden and sometimes suppressed in weighing the epidemiological evidence.

An unexpected consequence of the coronavirus disease (COVID-19) pandemic has been to thrust the science of epidemiology into the spotlight as public health experts struggle to devise policies to combat a threat still poorly understood. The best way to interpret the vast variety of freshly generated findings in a context of scanty information on virus behavior and evolution has confounded experts and laypersons alike, often complicated by the admixture of commercial and political factors that can perturb and/or distort interpretations. This Journal and environmental epidemiology in general are well acquainted with the problems, although they may be less publicly visible and often not appreciated by policy makers, the media and laypersons.

A principal method to deflect unwanted policy implications of properly conducted epidemiological studies is to deliberately frame the results in a way that casts doubt and manufactures uncertainty about their validity [1, 2]. We use the word “manufacture” to emphasize the recognizable techniques employed by special interests to accomplish this. We have previously covered various aspects of the problem in the Journal [3, 4], and we will continue to raise this issue because of its paramount importance to scientists, policy makers and the public. The article by Soskolne et al. just published in *Environmental Health* [5] amply illustrates the many approaches to generating doubt. Controversies about the interpretation of the research studies, as well as their policy implications, are being amplified by what the World Health Organization (WHO) has called an *infodemic* of distortion and untruths [6].

Thoughtful and constructive critique is a necessary part of science, of course. This Journal practices open peer review to facilitate the exchange of frank and transparent views and perspectives among colleagues in the field [7]. The openness is a recognition that where one stands often depends upon where one sits, as the cliché goes. The goal is to weed out purposefully distorted interpretations produced by financial or other sources of conflicting interests. The techniques employed can be subtle and hard to recognize. They often seem to appeal to science’s best aspirations. For example, “sound science” and “evidence-based toxicology” have been employed to counter findings that have the potential to be troublesome to a well-resourced or situated special interest [8, 9]. This approach has even taken the form of a call for “Good Epidemiological Practice” that originated with industry groups [10] whose apparent goal was to discredit evidence that was considered unwelcome.

For example, a frequent strategy has been to elevate Bradford Hill’s well-known general viewpoints on features of causal associations to necessary and sufficient criteria, although the author never used the word “criterion,” but referred to aspects and viewpoints, while emphasizing his nine “examinations” as useful “if available and applicable” [11]. Thus, the nine features should not be employed as a checklist, as causality cannot be established by satisfying a simple list of qualitative conditions [12–14]. Further, the aspects of causal associations are asymmetric: Although affirmative answers may support causality, none of them is a necessary condition (perhaps apart from the temporal relationship), and the lack of one or more affirmative answers does not speak against causation [15].

A consequence of these tactics, as described in the “Late lessons from early warnings” project of the European Environment Agency [16] and also highlighted in this Journal [4], many optimistic assumptions on supposedly innocuous chemicals were later found to be misleading and in fact dangerous to human health. That a chemical is innocuous unless scientific documentation has shown otherwise has been appropriately named the “untested chemical assumption” by a committee of the U.S. National Research Council [[Bibr CR17]].

The controversies are magnified by the ongoing infodemic that rapidly disseminates all kinds of health information along with half-truths through a variety of media and informational channels [6]. Because of concerns about misinformation and resulting chaos in relation to the COVID-19 pandemic, WHO has called for better management of online platforms and for building resilience to the distorting effects of misinformation. These recommendations follow a thorough report from a working group on how to end the infodemic [18], in which transparency and prevention of conflicts of interest feature prominently.

Unlike online social media platforms, scientific journals have not been identified as primary contributors to the infodemic associated with the coronavirus pandemic. Most journals, like the present one [7], comply with the guidelines of the Committee on Publication Ethics and require disclosure of potential conflicts of interest (https://publicationethics.org). Simple disclosure, however, is a necessary but not a sufficient step to resolve conflicts of interest. The International Network for Epidemiology in Policy (INEP) responded to this situation by releasing an extensive Position Statement on Conflict-of-Interest and Disclosure in Epidemiology (https://epidemiologyinpolicy.org/coi-d-position-statement). Drawing from this source, a new *Environmental Health* article [5] expands on, explains, and provides literature references to each of 33 tactics, most of them from the initial version of the toolkit in the INEP statement. These items constitute methods/techniques, arguments, and other tactics commonly used to distort and misapply epidemiological science.

That management of conflicting interests in science may be threatened by vested interests is exemplified by a recent issue of the *American Journal of Health Behavior* on the use of electronic nicotine delivery devises among adults [19]. Every article in the issue was authored by scientists employed by JUUL (a manufacturer of electronic cigarettes) or authors hired as consultants to the tobacco industry. Peer review was reportedly conducted, but, in the absence of open peer review, the identity and affiliations of the peer reviewers are unknown. The special issue was released at a time where JUUL’s application to continue sales is under consideration by the U.S. Food and Drug Administration. This incident suggests that the infodemic is also affecting scientific publication, as has also occurred in the past with, e.g., the food industry [20].

Consideration of study validity and scrutiny of scientific inference and conclusions are crucial for scientific journals, along with transparency and appropriate handling of potentially conflicting interests. The range of possible (and impossible) forms of critique that may be raised on behalf of vested interests or other groups who desire to blunt or obscure the thrust of the epidemiology conclusions is substantial [5]. The new toolkit may serve as a checklist of, or guide to key methodological issues, to identify whether epidemiology results are being or could be misinterpreted, and for use by those who seek better ways to teach critical review of the scientific literature. Although epidemiologists are often skillful “flaw catchers”, this toolkit may be particularly valuable for training epidemiologists and other healthcare professionals on the ways in which epidemiology can be distorted. In this so-called post-truth era and with the advent of the infodemic, there is an increasingly urgent need for additional safeguards to protect research integrity.

Given the confusing claims on how to interpret epidemiology research, the article by Soskolne et al. [5] is particularly welcome in helping to sort out which are genuine uncertainties or conundrums and which are manufactured.

## Abbreviations

COVID-19: Coronavirus disease 2019
INEP: International network for epidemiology in policy
WHO: World Health Organization
